# Causes of Death in Stray Cat Colonies of Milan: A Five-Year Report

**DOI:** 10.3390/ani11113308

**Published:** 2021-11-19

**Authors:** Valeria Grieco, Paola Crepaldi, Chiara Giudice, Paola Roccabianca, Giuseppe Sironi, Eleonora Brambilla, Sonia Magistrelli, Giuliano Ravasio, Federico Granatiero, Anna Invernizzi, Mario Caniatti

**Affiliations:** 1 Department of Veterinary Medicine (DIMEVET), University of Milan, Via dell’ Università 6, 26900 Lodi, Italy; valeria.grieco@unimi.it (V.G.); paola.roccabianca@unimi.it (P.R.); giuseppe.sironi@unimi.it (G.S.); eleonora.brambilla@unimi.it (E.B.); giuliano.ravasio@unimi.it (G.R.); mario.caniatti@unimi.it (M.C.); 2 Department of Agricultural and Environmental Sciences-Production, Landscape, Agroenergy (DiSAA), University of Milan, Via Celoria 2, 20133 Milano, Italy; paola.crepaldi@unimi.it; 3 Agenzia della Tutela della Salute (ATS) città Metropolitana di Milano, Dipartimento Veterinario, Via Amendola 3, 20090 Milano, Italy; smagistrelli@ats-milano.it; 4 Clinica Veterinaria Prealpi, Via Monviso 2, 22070 Limido Comasco, Italy; info@prealpivet.it; 5 Istituto Zooprofilattico Sperimentale della Lombardia e dell’Emilia Romagna (IZSLER), Via Giovanni Celoria 12, 20133 Milano, Italy; anna.invernizzi@izsler.it

**Keywords:** cat, colony cats, stray cats, causes of death, trauma, feline panleukopenia, feline infectious peritonitis, renal failure, parasites

## Abstract

**Simple Summary:**

Cats have been closely linked to humans for thousands of years. Nowadays, stray cats are frequently hosted in colonies, protected, and enrolled in programs of trap–neuter-–return to control population increase. Italian public veterinary services work in collaboration with voluntary colony caretakers and are responsible for neutering and monitoring the health of colony cats. This retrospective study, conducted by the Anatomical Pathology Unit of the Teaching Veterinary Hospital of Milan in collaboration with the public veterinary services, was undertaken because of the limited information available regarding causes of death of colony cats. The study reports on and statistically analyzes the causes of death of colony cats in the city of Milan as assessed by necropsy. Inflammatory processes including those consistent with the most relevant feline infectious diseases were most common in kittens and young cats. Trauma was more frequent in adult cats, while organ failure was the most common cause of death in aged cats. Considering the possible animal welfare issues deriving from colony cats, awareness of the most common causes of death and collaboration between university veterinary pathologists and public veterinary services represent an essential contribution to health monitoring of colony cats and can assist in the rapid detection of possible emerging animal welfare concerns.

**Abstract:**

The presence of cats in urban environments has a long history. In Italy, stray cats are protected by national and regional laws, and programs of neutering and reintroduction to colonies are ongoing. Colony cats have been widely studied from a behavioral perspective, while surveys regarding their causes of death are limited, although they may provide relevant information related to public health and cat welfare. This retrospective study provides pathological descriptions and statistical analyses of the causes of death of 186 cats from 100 colonies in the city of Milan. Inflammatory processes represent the primary cause of death (37.7%) and include common feline infectious diseases such as feline panleukopenia (67.5%), particularly in kittens, and feline infectious peritonitis (32.5%), most common in adult cats. Trauma was found to be a common cause of death of young/adult cats (14%) with a generally good body condition, while severe parasitosis was less represented (2.6%). The death of old cats was statistically associated with organ failure (24.7%), particularly renal failure, and tumors (11.8%). Knowledge of the most common causes of death of colony cats could make an important contribution to the health monitoring of these cats and sanitary control of their habitats and provide information on possible related emerging animal welfare concerns.

## 1. Introduction

Populations of unowned cats exist throughout the world [1], and cats have been closely linked to human society for thousands of years [2]. The most ancient evidence of cat–human co-existence, dating to 7000 years BC, was discovered in Cyprus, followed by Egyptian evidence dating to 2000 years BC [3,4]. In Italy, the presence of domestic cats in urban environments has a long history, with ancient Roman documents reporting stray cats living around monuments and in public gardens [5]. Many public issues related to stray cats include health concerns with regard to zoonotic diseases, the spread of diseases to pet cats, public nuisance, and the welfare of the cats themselves [2,6,7]. Stray cats are considered a threat to native wildlife, but when they are grouped in managed colonies, studies have revealed minimal bird predation by colony cats [8]. A cat colony is nowadays mainly defined as a group of three or more sexually mature cats living and feeding close to one another [9]. Cat colonies are not mere aggregations of individuals around sources of food, but represent truly structured and functional social groups [10,11], even if a consistent source of adequate food provided by caretakers is essential for a colony to remain in one location [2].

Italy signed and ratified the European Convention for the Protection of Pet Animals and delegates regions to organize birth control [12], protecting stray cats in feline colonies that are regularly counted and registered. Recent data refer to around 70,000 colonies of cats, 11,000 of which are in Lombardy, the region where Milan is the main town. In Milan, approximately 1000 cat colonies have been registered by the veterinary public service (VPS). Cat colonies are provided with food and shelter by caretakers, who also participate in the no-killing, trap–neuter–return (TNR) program. Since 1991, TNR is the only legal policy in Italy for limiting the cat population [13]. With TNR, veterinarians identify cats with microchips, and their neutering status is indicated by a small cut on the ear. Caretakers play a fundamental role in the TNR process for the welfare and health management of cats by alerting municipal veterinarians in case of cat illness or death. The Veterinary Teaching Hospital (VTH) of the University of Milan is involved in TNR and the health management of colony cats through an agreement signed with the VPS (ATS Città metropolitana di Milano). The VTH provides hospitalization for severely ill or traumatized cats, and allows submission of dead cats from registered colonies for necropsy. Regular post-mortem examinations of colony cats, performed at the pathology unit of the VTH, makes a significant contribution to the health monitoring of the cat population in this urban territory.

Several studies on unowned cats have been published in the last three decades, however, most of these have mainly focused on either the TNR program [1,7,14], the complete eradication of stray cat populations in specific areas [15,16], or an analysis of the prevalence of single infectious/parasitic diseases in certain regions or groups of colonies [17,18,19]. On the other hand, causes of death of colony cats have only been occasionally mentioned [6,20,21]. In a recent paper focusing on the possible negative effect of stray cats on bird populations, the stomach contents of cats were analyzed, but the causes of death of the examined cats were not reported [22].

The aim of the present study is to report on and critically and statistically analyze the causes of death of colony cats in registered colonies in the municipality of Milan.

## 2. Materials and Methods

No animals were killed for this retrospective study. Necropsies of the colony cats were performed as part of the health monitoring programs according to the agreement between the Veterinary Public Service of the city of Milan and the VTH/Pathology Unit.

### 2.1. Animals

For the present retrospective study, the archives of the VTH Pathology Unit were searched for colony cats submitted for necropsy by the municipal VPS between 2014 and 2018.

For each cat, together with gross and histological findings, the following data were mostly reported: breed, age, sex, colony of origin, body condition, and cause of death. The age was estimated at necropsy and in the database was reported as kitten, young, adult, or old. Body condition evaluated at necropsy was recorded as emaciated, very thin, underweight, ideal, overweight, or obese. The town colony location was defined as central, middle, or peripheral, according to Abakumova (2013) [23].

Causes of death were grouped as follows: (1) trauma; (2) inflammatory process (infectious and non-infectious diseases); (3) organ failure (kidney, liver, heart); (4) tumors, (5) severe parasitic disease; and (6) pulmonary edema (with no other apparent cause). For causes of death of single cats, a seventh group was created, named “miscellaneous causes”, which included all causes that were observed either occasionally or in single individuals. The eighth group included cases for which the cause of death remained undetermined.

### 2.2. Statistical Analysis

Descriptive statistics, chi-square, and Fisher’s exact test were used to describe the causes of death and the associations with the different recorded categorical factors: age, sex, colony of origin, and body condition. Statistical analysis was performed using JMP^®^ 13.0.0 (SAS, 2016, Cary, NC, USA).

In addition, logistic regression analysis was performed on a subsample consisting of the 89/186 cats for which information related to all categorical factors considered in the study were available in the database. In the logistic regression model, cause of death as the dependent variable using age, sex, neutered status, and body condition, and colony location as the independent variable were analyzed.

## 3. Results

### 3.1. Cats

Necropsy records from 186 cats were retrieved from the electronic archives (Table S1, Supplementary Material).

### 3.2. Colonies

The location of the colony was reported for 98 out of 186 cats (52.69%). For 10 of these colonies, multiple cats (two to five) were submitted for necropsy. The town distribution of colonies was central for 7/98 (7.1%), middle for 10/98 (10.2%), and peripheral for 81/98 (82.7%).

### 3.3. Breeds and Sex

All cats were domestic shorthair breed, and the sex was reported in 181 cases: 92 were male and 89 female. Eighty cats (80/181) were neutered, 43 (53.75%) male, and 37 (46.25%) female.

### 3.4. Age

The age was indicated for 170 cases: 32 (18.8%) were kittens, 24 (14.2%) were young, 44 (25.88) were adult, and 70 (42.1%) were old cats. The approximate age was not reported for 16 cats.

### 3.5. Body Condition

Body condition was reported for 180 out of 186 cats: 47 of the 180 were in ideal body condition, 25 were underweight, 11 were overweight, 22 were emaciated, and 75 were very thin.

### 3.6. Causes of Death

**Trauma**. Traumatic lesions were recorded for 27 out of 186 cats (14%); 12 were male and 15 female. Considering the age distribution, traumatic injury was the cause of death for three kittens, seven young, 11 adult, and two old cats. Age was not reported for eight cats. Body condition was reported for 26/27 traumatized cats. No cat was emaciated, seven were very thin, two were underweight, 13 were in ideal body condition, and four were overweight. Lesions observed included large hemorrhagic suffusions/hematomas, alone or associated with severe muscle ruptures and/or multiple bone fractures, frequently exposed or comminuted. The most commonly injured areas were the dorsal and hip regions and the hind legs. In one case, a diaphragmatic hernia was diagnosed.

**Inflammatory processes**. Severe inflammatory disease was the cause of death for 70/186 cats (37.7%): 30 male, 39 female, and in one for which sex was not reported. Age was reported for 64/70 cats: 26 were kittens, 10 were young, 12 were adult, and 16 were old. In 40/70 cats, gross lesions observed at necropsy were consistent with either feline panleukopenia (feline infectious enteritis) (27/40) or feline infectious peritonitis (13/40). These diagnoses were histopathologically confirmed.

**Figure 1:**
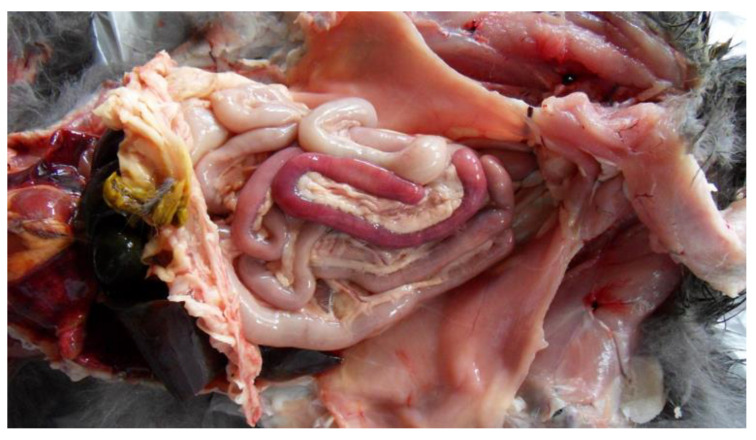
Cat abdominal cavity. Severe hyperemia of small intestine, jejunum, and ileum associated with edema of mesenteric lymph nodes.

Feline panleukopenia (FP) was diagnosed in 27/40 cats (67.5%): 19 female and eight male; 19 were kittens, five were young, and for three cats, the age was not reported. Body condition was reported in 25/27 of FP cases: 15 cats were very thin, five were underweight, and five were in ideal body condition. Grossly, severe hyperemia, and hemorrhagic enteritis of small intestine, jejunum, and ileum associated with edema of the mesenteric lymph nodes was observed in all cases (Figure 1). Histologically, crypt epithelial attenuation/necrosis with crypt ectasia or loss, villous atrophy, and collapse were observed in the small intestine. Peyer’s patches were severely depleted or regenerative depending on the stage of the disease. Edema and follicular depletion were detected in mesenteric lymph nodes. Lymphocytic depletion was also observed in the spleen and thymus.

**Figure 2:**
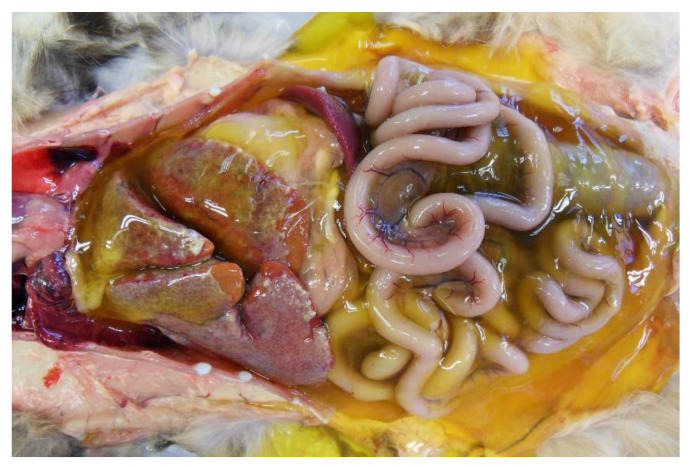
Cat abdominal cavity. Yellowish effusion filled peritoneal cavity. Surfaces of abdominal structures are covered with plaques of white granular material (fibrin).

Feline infectious peritonitis (FIP) was diagnosed in 13/40 cases (32.5%): five cats were female, seven were male, and the sex was not recorded for one cat. Age was reported in 12 cases: one kitten, one young, five adult, and five old cats; three cats were emaciated, one was underweight and two overweight, four were very thin, and three were in ideal body condition. Five cats were affected by non-effusive (dry form) FIP. In these cases, histopathology identified pyogranulomatous lesions in various organs, with typical vasculitis and perivasculitis, especially of small to medium-sized venules of the renal cortex, lungs, and liver. Macrophages predominated and were associated with varying numbers of neutrophils, lymphocytes, and plasma cells. In 3/5 cats, only the abdominal organs were affected, while in two catsm both abdominal and thoracic organs were affected. Eight cats had effusive FIP (wet form). In seven cats, peritoneal effusion was observed, and in one cat, pleural effusion was also present. In all cases (8/8), an abundant, viscous, clear, and pale to deep yellow exudate, occasionally containing strands of fibrin, was documented (Figure 2). Strands of fibrin adhering to the omentum and serosal surfaces were also present. Pyogranulomatous lesions were occasionally seen in the parietal peritoneum, in the omentum, and on the surface of the main abdominal organs. In one case, pyogranulomas affected the visceral pleura and mediastinal lymph node.

Pneumonia was reported in 19 cats, 10 male and nine female. Body condition was not reported for one cat, four cats were emaciated, three were underweight, eight were very thin, and three were in ideal body condition. Age was not reported in one case, five cats were old, six were adult, three were young, and four were kittens. Lungs were overall increased in volume and weight, with altered consistency. Suppurative pneumonia was most commonly diagnosed and was grossly characterized by hyperemic and irregular pulmonary surface with fibrous thickening of the interlobular septa. Areas of consolidated parenchyma were visible, together with gray nodules scattered throughout, revealing purulent exudate on cut surfaces. Histologically, alveolar and bronchiolar lumina were filled with edematous fluid and numerous degenerate and non-degenerate neutrophils, a few mature lymphocytes and plasma cells, scattered foamy reactive macrophages, and scant necrotic debris; similar inflammatory infiltration was frequently observed within bronchial/bronchiolar walls and expanding alveolar septa.

Inflammatory processes other than pneumonia were diagnosed in 11 cats, six female and five male. In 10 cases, the age was reported: one adult, six old, two kittens, and one young cat. Regarding their weight, six of these cats were very thin, three were in ideal body condition, and two were underweight. Four cats were affected by enteritis: two of these four were old, one was young, and one was adult; three of the four were in poor body condition and one was underweight. Multifocal to coalescing facial subcutaneous abscesses were reported in two old cats.

**Organ failure**. Forty-six cats died because of severe organ failure involving one or multiple organs.

Renal failure due to chronic kidney disease was reported in 32 cases (32/46, 70%): 19 were male and 11 female, and sex was not recorded for two cats. Among these cats, 24 were old, seven were adults, and age was not reported for one; eight cats were emaciated, 16 were very thin, three were underweight, and five had ideal weight. Chronic renal failure was grossly associated with grayish, small, firm kidneys (end-stage kidneys) with multifocal to coalescing irregular depression of the surface. On cut surfaces, cortex was thinned, firm, and sclerotic. Histologically, interstitial fibrosis, glomerulosclerosis, atrophic nephrons, and ectatic tubules and dilated Bowman’s spaces were present. Multifocal, interstitial, severe lymphoplasmacytic inflammation was observed in most cases, while tubular mineralization was rarely observed.

Hepatic failure was reported in 6/46 cases (13%), four males and two females, two adults and four old cats. Cats were very thin in two cases. In one case, the body condition was not reported, one was emaciated, one was underweight, and one was in ideal body condition. Cats were frequently icteric. Grossly, the liver was enlarged, greasy, and fragile. The liver was yellow in four cases histologically characterized by severe diffuse macrovescicular intrahepatocytic lipidosis (fatty degeneration). In another case, the liver was pale brownish and histologically characterized by interstitial deposition of eosinophilic amorphous homogenous material that stained positive with Congo Red (amyloidosis). In the sixth case, the liver was reduced and firm, with an irregular surface. Microscopically, lobules of degenerated hepatocytes were separated by septa of fibrous connective tissue containing multiple proliferating small bile ducts (cirrhosis).

Pancreatic failure was recorded in one very thin, old male cat. In this case, the pancreas was reduced in size, firm, and pale. In addition, small multifocal dry yellowish plaques were detected on the surrounding mesentery (fat necrosis). Histologically, severe diffuse fibrosis of the interstitial pancreatic septa was detected.

Heart failure was reported in five cats, four male and one female generally in good body condition. In these cases, severe concentric cardiac hypertrophy with reduced left ventricular lumen was observed. Ventricular myocardial interstitial fibrosis was histologically detectable in two cases.

Multiorgan failure was observed in two old female cats. One was emaciated and showed end-stage kidney disease, severe diffuse hepatic degeneration, and left myocardial hypertrophy. The other cat was very thin, and pancreatic and severe hepatic fibrosis were detected.

**Tumors.** Neoplastic lesions were observed in 22 cats, 12 female and eight male (in two cases, sex was not recorded). The age of two cats was unknown, 15 were old, three were adult, and two were young. Four cats were emaciated, five underweight, four were in ideal body condition, and six were very thin. The most common tumors were squamous cell carcinoma (6/22 cases) and lymphoma (5/22 cases). Two cats were diagnosed with osteosarcoma, while in the other cats, the following tumors were diagnosed: feline injection site sarcoma, hepatic carcinoma, pancreatic carcinoma, pulmonary carcinoma, ceruminous gland adenocarcinoma, histiocytic sarcoma, oral fibrosarcoma, myeloid leukemia, and thyroid adenocarcinoma.

**Parasitosis.** Parasites were reported as the cause of death in five cats, four male and one female; one was adult, one was young, and one was a kitten, and the age of the fifth was unknown. Three of these cats were very thin, one was underweight, and for one, the body condition was not reported. Three cats were affected by *Aelurostrongylus abstrusus*. In these cases, multiple consolidated pulmonary areas and small nodules protruding on the lung surface and scattered in the parenchyma were grossly observed. Histologically, eggs and larvae in various stages were observed in both the alveoli and bronchioles, surrounded by eosinophilic and granulomatous reactions. One cat showed the massive presence of nematodes (*Toxocara cati*) in the intestinal lumen. In the other case, hookworms, morphologically consistent with Ancylostomadidae, were detected in the small intestine.

**Pulmonary edema.** This was the only lesion observed in 10 cats, six male and four female. The age was reported in nine cases: four were adult, two were old, two were young, and one was a kitten. Body condition was ideal in four cats, three cats were very thin, one cat was underweight, one emaciated, and for one cat, it was not recorded.

**Miscellaneous causes of death.** Three male cats were classified in this category. In two cats, cardiovascular collapse was suspected, while one cat was affected by ischiatic thrombosis.

**Indeterminate causes of death.** For one old male cat and a female kitten in advanced putrefaction, the cause of death remained indeterminate.

### 3.7. Statistical Analysis

The distribution and interaction of the variables for the cats (sex, age, and body condition) with causes of death were analyzed with the chi-square test and logistic regression, as described in the Materials and Methods.

**Figure 3:**
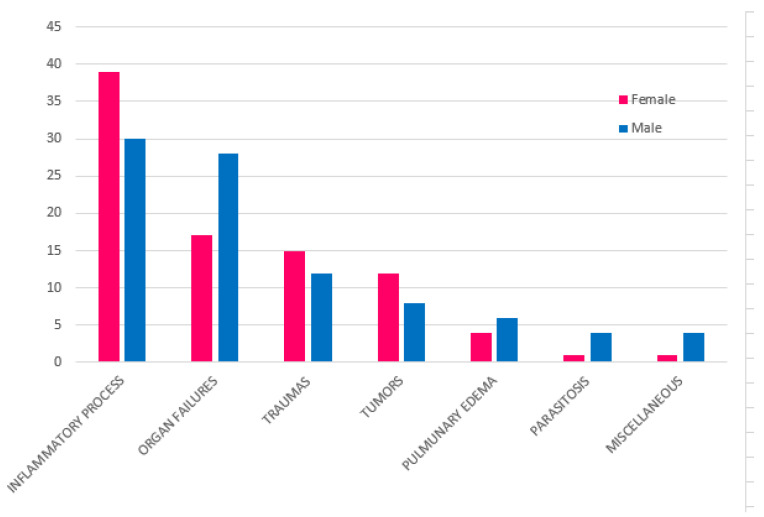
Relation between cause of death and sex of colony cats included in the study.

**Figure 4:**
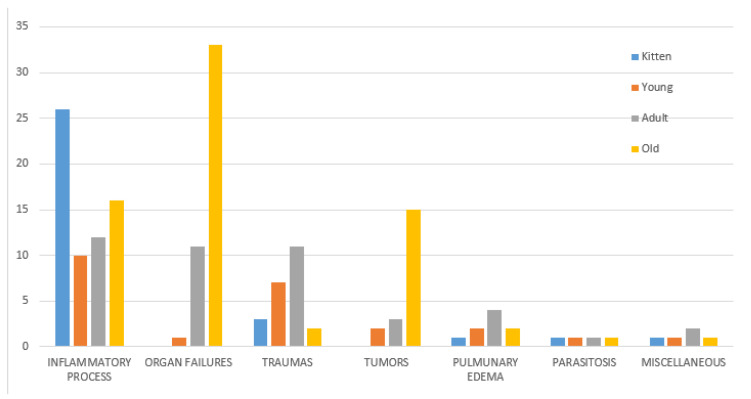
Relation between cause of death and age of colony cats included in the study.

**Figure 5:**
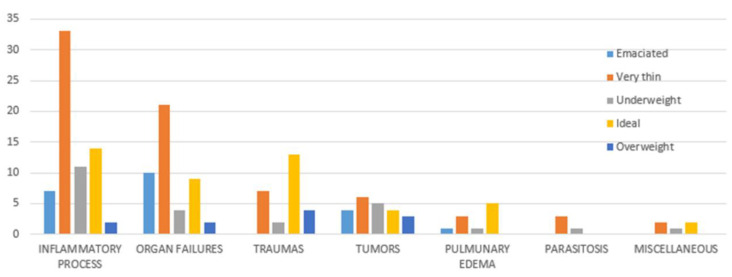
Relation between cause of death and body condition of colony cats included in the study.

No statistical differences were observed between male and female cats by the chi-square test. There were statistically significantly (*p* < 0.05) fewer neutered cats (*n* = 80, 44%) than non-neutered ones (*n* = 100, 56%), however, the neutered rate increased with age, reaching 56 and 66% in adult and old cats, respectively. No statistically significant differences between the sexes with regard to neutering was observed in the sample analyzed. A significant difference (*p* < 0.05) was observed between sex and age, with a larger number of dead male adult cats (*n* = 30, 68%). Cause of death was not significantly related to sex (Figure 1), although the higher impact of organ failure as a cause of death in males was evident (Figure 3). A significant difference (*p* < 0.001) was observed between age and causes of death (Figure 4). Specifically, inflammatory processes were the main statistically significant cause of death for kittens (81%), and the most common cause of death for 59% of young and adult cats, even if not significant. The main cause of death in old cats was organ failure (55%), followed by tumors (25%). As expected, organ failure was negatively associated with young age; indeed, none of the young cats died from organ failure. Death of young and adult cats was significantly associated with trauma, which was involved in 21 and 25% of recorded deaths, respectively. Within organ failure, renal failure was the most prevalent cause of death at 67%, significantly represented by neutered old male cats. A statistically significant (*p* = 0.002) association between body condition and cause of death was also observed (Figure 5). Specifically, ideal and overweight cats were significantly more likely to die of traumatic events. Conversely, emaciated cats were frequently affected by organ failure, although the chi-square test was not significant.

**Figure 6:**
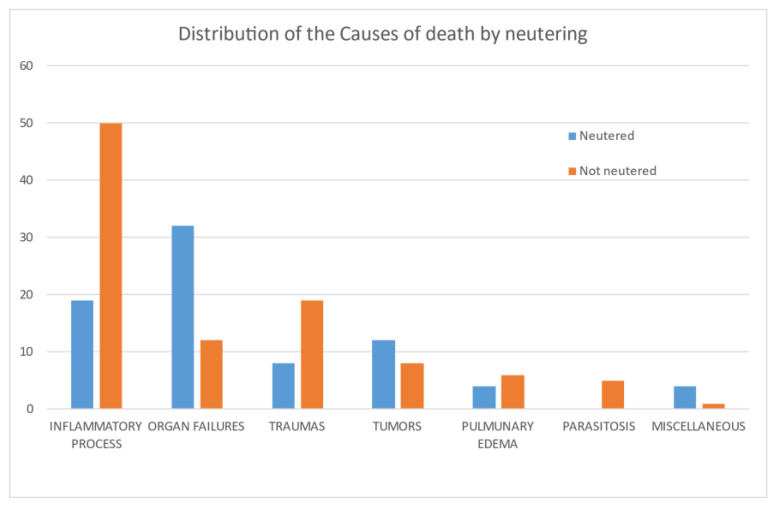
Relation between cause of death and neutering of colony cats included in the study.

The logistic regression model (R^2^ = 0.397, *n* = 87), similar to the chi-square test, showed that only age (*p* = 0.00467) and body condition (*p* = 0.0209) were statistically significant. No statistical effect was observed for neutering with this model, even if a statistically significant difference was observed with the chi-square test considering only the interaction between cause of death and neutering. This result is mainly associated with a confounding effect due to the different ages of non-neutered and neutered cats. In fact, as reported in Figure 6, cats that died from inflammatory processes were mainly kittens (non-neutered), and cats affected by organ failure were frequently old and thus generally neutered.

## 4. Discussion

The presence of stray cats in the urban environment has long been known. In Italy, stray cats are protected by national and regional laws [24]. In particular, national law No. 281 (1991) has two key points: stray cats have the right to live undisturbed freely, gathered in colonies, and they must be neutered by the local veterinary public service (VPS) and then reintroduced to their colony. In addition, colony cat caretakers become institutionalized figures who gather in associations, and can obtain the official assignment of managing a cat colony. Feeding, trapping, neutering, and releasing stray cats and allowing them to live in colonies is an answer to the overpopulation problem [6], and TNR programs are considered the most practical, effective, and humane way to control stray cats [1,13]. The finding of a substantial representation of old cats in the current study vouches for the success of the monitoring provided by VPS and caretakers. In addition, collaboration between VPS and the VTH plays an important role in monitoring cats’ health [24,25].

Because of the scarce literature, the present retrospective study was aimed at reporting and statistically analyzing the causes of death of colony cats submitted for necropsy in the urban environment of Milan.

Although it must be considered that cats can move from colonies and die far from them with no possibility of being monitored, the number of cats retrieved from the database was representative of the colony cat population supervised by VPS.

As expected, most cats (83%) in this study came from colonies located at the town periphery, where there are green spaces and old abandoned buildings and colonies are more tolerated. Unfortunately, we had data on the colony location for just half of the cats considered. We suggest that it is important to register colony locations in order to investigate how social differences might correlate not only with cause of death, but also with animal welfare.

A previous study regarding cat colonies in Rome [10] reported no prevalence of one sex over the other, while an Australian study reported a prevalence of male cats [26]. In the present study, there were significantly fewer neutered cats than non-neutered ones. This was probably due to the large number of kittens and young cats, as statistics revealed that the neutered rate increased with age, reaching 56 and 66% in adult and old cats, respectively. These findings are in line with the VPS policy of neutering at a minimum age of four months. In addition, the constant availability of food and care attracts stray cats [6,13], therefore, the presence of a certain percentage of newly entered, non-registered, and non-neutered cats must also be taken into account. Moreover, it must be also noted that one of the most relevant reported problems concerning the reduced success of TNR seems to be that the continuing increase in the number of cats because of the presence of colonies, where cats are sustained and monitored, can “morally justify” the abandonment of cats [2,6,27,28].

Regarding the body condition, 46% of cats were overweight, slightly underweight, or in ideal body condition, while 54% were emaciated or very thin. Of note, 75% of the colony cats included in this study were affected by inflammatory diseases, organ failure, neoplastic disease, and parasitic diseases, which have been reported as the main causes of weight loss.

Concerning causes of death, inflammatory processes such as pneumonia and feline infectious viral diseases were the most frequent in the cat population examined (70/186). Severe pneumonia was detected in 19/70 cats and was not significantly associated with age, being diagnosed in kittens as well as young, adult, and old cats. Colony cats are exposed to climate factors and pathogens, which can be predisposing factors for pneumonia. Moreover, colony cats can contract respiratory diseases that in owned cats are prevented by routine vaccination. Pneumonia is one of the most common causes of death in cats. Viral pneumonia can be caused by felid herpesvirus-1 or feline calicivirus (FCV) infection [29] and can be complicated by bacterial superinfection [29,30]. In addition, in cats, primary bacterial suppurative pneumonia/bronchopneumonia is also frequently caused by *Bordetella bronchiseptica* [29] or other agents such as *Pasteurella* and *Mycoplasma* spp. [31,32]. Colony cats can also be affected by agents such as feline leukemia virus (FELV) and feline immunodeficiency virus (FIV), ref. [28] which compromise the immune system, predisposing the animals to infection.

Other inflammatory processes occurred in 40/70 cats examined and were consistent with common feline infectious diseases such as FP and FIP.

In the present study, FP was significantly more common in kittens and young cats, in agreement with other reports describing FP as a highly contagious and lethal disease that is less frequent in adult cats [33,34]. FP virus transmission occurs by orofecal or indirect contact, and due to its long persistence (one year or more), the contaminated environment could represent a source of infection for both colony and owned cats [22].

Another common feline infectious disease observed in the present study was FIP, a progressive fatal disease caused by feline coronavirus (FCoV) infection [35], diagnosed in 13 cats. Similar to the current literature, cats affected by FIP in this study showed severe systemic inflammation of serosal membranes and widespread pyogranulomatous lesions [36]. FIP tends to occur most frequently in cats < 2 years of age and less commonly in geriatric cats [35]. In our study, FIP cases also occurred in adult or aged cats. This is not surprising for colony cats, because, as reported for catteries/shelters, the incidence of FIP seems to be directly correlated with the number and density of cats. Moreover, stressors such as pregnancy, parturition, and surgical procedures such as spaying in TNR can increase the risk of developing the disease [37].

The second most common cause of death in the cats considered was organ failure, detected in 46/186 cats (25%). Organ failure was the main cause of death in old neutered male cats (67%), renal failure in particular (32/46). Although in other studies sex did not appear to be a factor related to the onset of chronic kidney disease, a strong prevalence of old cats, as in the present study, has been similarly described [38,39,40,41,42].

Renal failure is often associated with chronic kidney disease (CKD), which has been identified as an irreversible and progressive loss of renal function that occurs most commonly in geriatric domestic cats (>12 years of age) [43,44]. Generally, in CKD pathogenesis, a primary renal disease initiates parenchymal damage with loss of nephrons, followed by other factors intrinsic to the affected animal that lead to self-perpetuating renal injury [45]. Several primary renal diseases have been identified in cats including congenital disorders that have a typical breed predisposition [42,45]. All cats included in this study were domestic shorthaired, a breed not strictly predisposed to renal congenital disorders. On the other hand, it is important to consider that colony cats can develop other acquired renal disorders, since they are exposed to several risk factors including viral or bacterial infections and exposure to toxicants that may be present in the environment [45,46,47].

Other types of organ failure were less common in the present study (14/46). According to the literature [48], pancreatic failure is rare, and in this study was diagnosed in only one cat. Heart failure, generally related to hypertrophic cardiomyopathy (HCM), was reported in five cases. This low number was expected, since HCM is more common in specific breeds such as Maine Coon and Norwegian [49,50] than in shorthaired cats.

The second most prevalent cause of death in old cats was tumors (25%), consistent with the current literature reporting tumors as one of the main causes of death in aged cats [51]. The most common tumors were squamous cell carcinoma (six cases) and lymphoma (five cases), referred to in previous studies as the most common neoplastic diseases in cats [52,53,54]. The cause of most cutaneous SCC is chronic exposure to ultraviolet (UV) light, and in fact, these tumors are seen almost exclusively on the head, with white cats and colored cats with white areas being at greatest risk [52]. Lymphoma in cats commonly affects the gastrointestinal tract and seems to occur more frequently in cats seropositive for FIV and FELV, viruses that are easily transmitted among colony cats [53,54]. In the present study, only five cases of lymphoma were diagnosed, and this result is supported by the reduced seropositivity described in a recent study on colony cats of Milan (6.6% of cats positive for FIV, 3.8% positive for FELV) [55].

Regarding trauma as the cause of death, in the present study, young (21%) and adult male (25%) cats in ideal body condition were significantly more prone to dying from trauma than very thin and emaciated cats. Young and adult cats, fighting off various other infections/infestations, have more opportunity to die from trauma. Conversely, very thin and emaciated cats, most likely already affected by other diseases, tend to remain closer to the colony, coming into contact with fewer potential traumatic events.

Other studies have mentioned trauma as a common cause of death in kittens, which are reported to die or disappear from colonies within six months of birth [2,21]. In the present study, only three kittens died from traumatic lesions; these were more likely to die from infectious disease. However, traumatic death cannot be excluded for kittens that eventually disappear.

The least prevalent cause of death in the present study was parasitic disease. Pathological findings of verminous pneumonia due to *Aelurostrongylus abstrusus* were observed in three cats. *A. abstrusus* (Nematoda, Strongylida), a lungworm, is reported to be the most common lung parasite in cats worldwide [56,57]. Cats become infected by ingesting intermediate (snails) or paratenic (e.g., rodents, frogs, lizards, snakes, and birds) hosts [58,59]. Stray cats have greater exposure to intermediate and paratenic hosts, and actually, in the present study, all five cases of lung parasites were in colonies located at the extreme periphery of the town. In these areas, very close to the countryside, both intermediate and paratenic hosts are present, increasing the risk that colony cats will initiate the infection cycle. It must also be considered that *A. abstrusus* infection as well as other pulmonary diseases is cited as a risk factor for anesthetic-associated death at neutering [28,60].

Considering other possible causes of death in colony cats, there were no suspected cases of poisoning in the current caseload. In fact, as poisoning is a relevant public health concern, since 2008 in Italy, the carcasses of suspected poisoned animals must be referred for necropsy to specific veterinary public health centers named Zooprophlylactic Institutes, which rely on the Ministry of Health. Regarding Milan, during the period 2014–2018 considered in the present study, 11 carcasses of colony cats suspected of being poisoned were referred by the VPS to Istituto Zooprofilattico Sperimentale della Lombardia e dell’Emilia Romagna (IZSLER) and, in three of the 11, toxicological exams were positive for anticoagulant rodenticides. The use of anticoagulant rodenticides is common worldwide including in Italy [61,62]. Exposure occurs through ingestion of the rodenticide from bait containers or from the environment in which the rodents have carried the bait [63]. Relay toxicosis, which is intoxication by the ingestion of a previously poisoned animal, is unlikely to occur with rodenticide agents, because the amount in the rodent is small; however, it is possible in colony cats, as in other animals, that eventually prey upon rodents in areas where such bait is commonly used [63].

## 5. Conclusions

In conclusion, the present study shows that inflammatory processes (including those typical of common feline infectious diseases), traumatic lesions, organ failure, and tumors are the main causes of death among colony cats. Statistical analysis revealed a significant association between cause of death and age, with the most common causes of death in kittens and healthy young/adult cats being inflammatory processes and trauma, respectively. For older cats, the statistically significant causes of death were organ failure and tumors.

Since colony cats represent a possible animal welfare problem as well as a target, collaboration among caretakers, VPS, and veterinary faculties is important for monitoring them. In this scenario, knowledge of the most common causes of death could make an important contribution to the monitoring of cat populations and their environment and could alleviate emerging animal welfare concerns, allowing rapid intervention.

## Supplementary Materials

The following is available online at https://www.mdpi.com/article/10.3390/ani11113308/s1, Table S1: Data collected on 186 colony cats.
