# Supplementary material for: Causes of Death in Stray Cat Colonies of Milan: A Five-Year Report
Source: Animals (Basel). 2021 Nov 19;11(11):3308. doi: 10.3390/ani11113308 (PMC8614475; doi:10.3390/ani11113308)
Supplement: Supplementary file 1 [file animals-11-03308-s001.zip › animals-1469648-supplementary.pdf]

Table S1

Year	Protocol number	Sex	Neutering	Age	Cause of death	Body condition
**2015**	153	Male	Neutered	adult	other	ideal
**2016**	27	Male	Neutered	old	other	ideal
**2014**	104	Male	Neutered	young	other	ideal
**2016**	65	Male	Neutered	adult	other	underweight
**2016**	218	Male	Neutered	adult	other	poor
**2017**	205	Female		kitten	other	poor
**2015**	99	Female		young	pulmonary edema	poor
**2016**	86	Female		not recorded	pulmonary edema	ideal
**2016**	1	Female	Neutered	adult	pulmonary edema	ideal
**2017**	22	Male	Neutered	adult	pulmonary edema	ideal
**2016**	253	Male	Neutered	old	pulmonary edema	ideal
**2014**	231	Female	Neutered	young	pulmonary edema	ideal
**2016**	122	Male		adult	pulmonary edema	emaciated
**2014**	80	Male		adult	pulmonary edema	underweight
**2014**	48	Male		old	pulmonary edema	poor
**2018**	150	Male		kitten	pulmonary edema	poor
**2017**	148	Male		old	inflammatory processes	ideal
**2017**	50	Female	Neutered	old	inflammatory processes	poor
**2016**	257	Male		young	inflammatory processes	poor
**2016**	149	Female		kitten	inflammatory processes (enteritis)	underweight
**2016**	90	Male		adult	inflammatory processes (enteritis)	poor
**2017**	63	Female		old	inflammatory processes (enteritis)	poor
**2015**	104	Female	Neutered	old	inflammatory processes (enteritis)	poor
**2016**	128	Female	Neutered	old	inflammatory processes	underweight
**2016**	107	Female	Neutered	old	inflammatory processes	poor
**2017**	109	Male		kitten	inflammatory processes	ideal
**2015**	172	Male	Neutered	adult	inflammatory processes (pneumonia)	ideal
**2014**	177	Female	Neutered	adult	inflammatory processes (pneumonia)	
**2014**	271	Female		old	inflammatory processes (pneumonia)	emaciated
**2017**	156	Female		kitten	inflammatory processes (pneumonia)	ideal
**2017**	133	Male		young	inflammatory processes (pneumonia)	ideal
**2018**	80	Female	Neutered	old	inflammatory processes (pneumonia)	emaciated
**2017**	248	Male		adult	inflammatory processes (pneumonia)	emaciated
**2014**	54	Female	Neutered	old	inflammatory processes (pneumonia)	emaciated
**2016**	179	Female	Neutered	adult	inflammatory processes (pneumonia)	underweight
**2016**	184	Male	Neutered	adult	inflammatory processes (pneumonia)	underweight
**2016**	177	Male		kitten	inflammatory processes (pneumonia)	underweight

**2014**	66	Male		not recorded	inflammatory processes (pneumonia)	poor
**2017**	159	Male	Neutered	adult	inflammatory processes (pneumonia)	poor
**2018**	36	Female	Neutered	old	inflammatory processes (pneumonia)	poor
**2017**	54	Female	Neutered	old	inflammatory processes (pneumonia)	poor
**2017**	147	Male		kitten	inflammatory processes (pneumonia)	poor
**2016**	254	Male		kitten	inflammatory processes (pneumonia)	poor
**2018**	76	Female		young	inflammatory processes (pneumonia)	poor
**2017**	78	Male		young	inflammatory processes (pneumonia)	poor
**2015**	211	Male		not recorded	inflammatory processes (septicemia)	ideal
**2016**	213	Male	Neutered	adult	organ failure (heart)	ideal
**2017**	21	Female	Neutered	young	organ failure (heart)	ideal
**2015**	17	Male	Neutered	old	organ failure (heart)	overweight
**2014**	2	Male	Neutered	adult	organ failure (heart)	poor
**2015**	74	Male	Neutered	old	organ failure (heart)	poor
**2016**	222	Female		old	organ failure (liver)	ideal
**2017**	110	Male		adult	organ failure (liver)	emaciated
**2016**	125	Male		old	organ failure (liver)	poor
**2016**	21	Female	Neutered	old	organ failure (liver)	underweight
**2015**	128	Male	Neutered	old	organ failure (liver)	poor
**2016**	114	Male	Neutered	adult	organ failure (liver)	overweight
**2016**	35	Female	Neutered	old	organ failure (liver)	ideal
**2018**	151	Female		old	organ failure (liver)	emaciated
**2017**	179	Male	Neutered	old	organ failure (pancreas)	poor
**2018**	127	Male	Neutered	adult	organ failure (kidney)	poor
**2018**	30	Female	Neutered	old	organ failure (kidney)	poor
**2015**	42	Male	Neutered	old	organ failure (kidney)	poor
**2016**	52	Female	Neutered	adult	organ failure (kidney)	ideal
**2015**	52	Not recorded		old	organ failure (kidney)	ideal
**2018**	27	Male		old	organ failure (kidney)	ideal
**2017**	232	Male	Neutered	old	organ failure (kidney)	ideal
**2016**	68	Male	Neutered	old	organ failure (kidney)	ideal
**2017**	98	Male		adult	organ failure (kidney)	emaciated
**2017**	158	Female		old	organ failure (kidney)	emaciated
**2014**	251	Female		old	organ failure (kidney)	emaciated
**2018**	17	Female	Neutered	old	organ failure (kidney)	emaciated
**2017**	89	Male		old	organ failure (kidney)	emaciated
**2016**	170	Male		old	organ failure (kidney)	emaciated
**2016**	204	Male	Neutered	old	organ failure (kidney)	emaciated
**2016**	106	Male	Neutered	old	organ failure (kidney)	emaciated
**2018**	112	Male	Neutered	old	organ failure (kidney)	underweight
**2016**	28	Male	Neutered	adult	organ failure (kidney)	underweight
**2014**	14	Male	Neutered	old	organ failure (kidney)	underweight

**2018**	91	Not recorded		old	organ failure (kidney)	poor
**2018**	11	Female		not recorded	organ failure (kidney)	poor
**2017**	94	Female		adult	organ failure (kidney)	poor
**2015**	199	Female	Neutered	adult	organ failure (kidney)	poor
**2016**	126	Male	Neutered	adult	organ failure (kidney)	poor
**2018**	94	Female	Neutered	old	organ failure (kidney)	poor
**2017**	73	Female	Neutered	old	organ failure (kidney)	poor
**2017**	64	Female	Neutered	old	organ failure (kidney)	poor
**2017**	53	Male		old	organ failure (kidney)	poor
**2017**	206	Male	Neutered	old	organ failure (kidney)	poor
**2017**	113	Male	Neutered	old	organ failure (kidney)	poor
**2016**	152	Male	Neutered	old	organ failure (kidney)	poor
**2014**	51	Male	Neutered	old	organ failure (kidney)	poor
**2014**	211	Female		young	inflammatory processes (feline infectious peritonitis)	ideal
**2017**	129	Male	Neutered	adult	inflammatory processes (feline infectious peritonitis)	ideal
**2014**	212	Male	Neutered	old	inflammatory processes (feline infectious peritonitis)	ideal
**2016**	113	Male		adult	inflammatory processes (feline infectious peritonitis)	emaciated
**2015**	162	Male		old	inflammatory processes (feline infectious peritonitis)	emaciated
**2015**	95	Male		old	inflammatory processes (feline infectious peritonitis)	emaciated
**2018**	149	Female	Neutered	adult	inflammatory processes (feline infectious peritonitis)	underweight
**2018**	40	Not recorded		adult	inflammatory processes (feline infectious peritonitis)	overweight
**2014**	97	Female		old	inflammatory processes (feline infectious peritonitis)	overweight
**2017**	152	Male	Neutered	not recorded	inflammatory processes (feline infectious peritonitis)	poor
**2018**	37	Male	Neutered	adult	inflammatory processes (feline infectious peritonitis)	poor
**2014**	207	Female		old	inflammatory processes (feline infectious peritonitis)	poor
**2014**	210	Female		kitten	inflammatory processes (feline infectious peritonitis)	poor
**2014**	24	Female		not recorded	inflammatory processes (feline panleukopenia)	-
**2016**	234	Female	Neutered	not recorded	inflammatory processes (feline panleukopenia)	underweight
**2017**	211	Female		kitten	inflammatory processes (feline panleukopenia)	poor
**2018**	148	Male		kitten	inflammatory processes (feline panleukopenia)	-
**2017**	166	Male		kitten	inflammatory processes (feline panleukopenia)	poor
**2016**	248	Male		kitten	inflammatory processes (feline panleukopenia)	ideal
**2016**	180	Female		young	inflammatory processes (feline panleukopenia)	ideal
**2016**	229	Female		kitten	inflammatory processes (feline panleukopenia)	ideal

**2016**	16	Female		kitten	inflammatory processes (feline panleukopenia)	ideal
**2017**	242	Female		young	inflammatory processes (feline panleukopenia)	ideal
**2017**	139	Female		kitten	inflammatory processes (feline panleukopenia)	underweight
**2017**	227	Female	Neutered	kitten	inflammatory processes (feline panleukopenia)	underweight
**2016**	142	Male		kitten	inflammatory processes (feline panleukopenia)	underweight
**2015**	43	Female		young	inflammatory processes (feline panleukopenia)	underweight
**2017**	178	Female		not recorded	inflammatory processes (feline panleukopenia)	poor
**2017**	250	Female		kitten	inflammatory processes (feline panleukopenia)	poor
**2017**	231	Female		kitten	inflammatory processes (feline panleukopenia)	poor
**2017**	189	Female		kitten	inflammatory processes (feline panleukopenia)	poor
**2017**	188	Female		kitten	inflammatory processes (feline panleukopenia)	poor
**2017**	176	Female		kitten	inflammatory processes (feline panleukopenia)	poor
**2017**	175	Female		kitten	inflammatory processes (feline panleukopenia)	poor
**2016**	230	Female		kitten	inflammatory processes (feline panleukopenia)	poor
**2016**	219	Female		kitten	inflammatory processes (feline panleukopenia)	poor
**2017**	174	Male		kitten	inflammatory processes (feline panleukopenia)	poor
**2017**	142	Male		kitten	inflammatory processes (feline panleukopenia)	poor
**2016**	153	Male		young	inflammatory processes (feline panleukopenia)	poor
**2016**	147	Male		young	inflammatory processes (feline panleukopenia)	poor
**2015**	105	Not recorded		not recorded	neoplasia (lymphoma)	poor
**2015**	2	Female	Neutered	not recorded	neoplasia (squamous cell carcinoma)	-
**2018**	15	Male		adult	neoplasia (histiocytic sarcoma)	ideal
**2018**	77	Not recorded		old	neoplasia (osteosarcoma)	ideal
**2017**	155	Female		old	neoplasia (pulmonary adenocarcinoma)	ideal
**2016**	3	Female	Neutered	young	neoplasia (myeloid neoplasms)	ideal
**2018**	95	Male		adult	neoplasia (histiocytic sarcoma)	emaciated
**2014**	39	Female	Neutered	old	neoplasia (fibrosarcoma)	emaciated
**2016**	71	Male	Neutered	old	neoplasia (oral sarcoma)	emaciated
**2016**	256	Female	Neutered	young	neoplasia (lymphoma)	emaciated
**2017**	204	Female		old	neoplasia (squamous cell carcinoma)	underweight
**2017**	90	Female		old	neoplasia (squamous cell carcinoma)	underweight
**2015**	210	Female	Neutered	old	neoplasia (lymphoma)	underweight

**2018**	23	Male	Neutered	old	neoplasia (squamous cell carcinoma)	underweight
**2017**	80	Male	Neutered	old	neoplasia (squamous cell carcinoma)	underweight
**2014**	201	Female	Neutered	old	neoplasia (ceruminous gland adenocarcinoma)	overweight
**2017**	182	Male	Neutered	old	neoplasia (osteosarcoma)	overweight
**2015**	50	Male	Neutered	old	neoplasia (lymphoma)	overweight
**2015**	171	Female		not recorded	neoplasia (squamous cell carcinoma)	poor
**2018**	189	Female	Neutered	adult	neoplasia (squamous cell carcinoma)	poor
**2015**	98	Female		old	neoplasia (hepatocarcinoma)	poor
**2017**	33	Female	Neutered	old	neoplasia (lymphoma)	poor
**2014**	126	Male		old	neoplasia	poor
**2014**	41	Male		old	parasitosis	-
**2014**	167	Female		not recorded	parasitosis	underweight
**2017**	55	Male		adult	parasitosis	poor
**2015**	173	Male		kitten	parasitosis	poor
**2016**	203	Male		young	parasitosis	poor
**2017**	61	Male		adult	trauma	overweight
**2017**	245	Female	Neutered	not recorded	trauma	ideal
**2017**	183	Female		adult	trauma	poor
**2017**	267	Male		adult	trauma	ideal
**2017**	130	Male		kitten	trauma	underweight
**2015**	36	Female		young	trauma	ideal
**2014**	67	Female		young	trauma	-
**2018**	107	Female		not recorded	trauma	ideal
**2017**	99	Female		adult	trauma	ideal
**2016**	250	Female	Neutered	not recorded	trauma	ideal
**2017**	151	Male	Neutered	adult	trauma	ideal
**2016**	121	Female		adult	trauma	ideal
**2017**	135	Female	Neutered	adult	trauma	ideal
**2017**	76	Male		adult	trauma	ideal
**2017**	117	Female		kitten	trauma	ideal
**2015**	75	Female		young	trauma	ideal
**2016**	63	Female		young	trauma	underweight
**2018**	1	Male		not recorded	trauma	overweight
**2017**	36	Male		adult	trauma	overweight
**2017**	190	Male	Neutered	young	trauma	overweight
**2016**	115	Female		adult	trauma	poor
**2018**	124	Female	Neutered	old	trauma	poor
**2017**	108	Male	Neutered	old	trauma	poor
**2016**	187	Male		kitten	trauma	poor
**2017**	184	Female		young	trauma	poor
**2015**	100	Male		young	trauma	poor
